# Voice, vulnerability, and expressive growth: investigating AI anxiety and performance appraisal in voice arts education

**DOI:** 10.3389/fpsyg.2025.1653502

**Published:** 2025-11-13

**Authors:** Danna Ouyang

**Affiliations:** School of Communication, Fujian Normal University, Fuzhou, China

**Keywords:** artificial intelligence anxiety, voice performance education, expressive self-assessment, project-based learning, educational psychology.

## Abstract

As artificial intelligence (AI) voice technologies rapidly evolve, students training in vocal performance increasingly grapple with anxiety about their artistic identities and career trajectories. This study investigates how AI-related Anxiety (AIA) influences both self-perceived artistic expressiveness (SAE) and external evaluations of expressive performance (TAE). Anchored in the Vienna Integrated Model of Art Perception (VIMAP), and employing a revised version of the Expressive Power Scale, we conducted a mixed-methods study involving 414 vocal performance students and 414 independent raters across three Chinese universities. Quantitative analyses revealed that students with heightened AIA tended to undervalue their expressive capacities, even when third party ratings were favorable. Structural equation modeling indicated that AIA negatively predicted externally assessed expressiveness, and self-assessment had only a weak positive effect. In the qualitative findings, novel psychological themes were identified including emotional distancing and rediscovering one’s creative agency in the face of AI. These results underline the significance of integrating both psychological skills and AI literacy into vocal performance training, while also ensuring students can retain their artistic identity in a potentially AI-driven context.

## 1 Introduction

The rapid advancement of artificial intelligence (AI) in voice synthesis is reshaping the landscape of vocal performance, raising urgent concerns about technological displacement within the creative arts (Ghayem, 2024). With state-of-the-art text-to-speech (TTS) systems and virtual anchoring technologies now capable of replicating human vocal nuances with striking accuracy, studies show that audiences often struggle to differentiate between AI-generated and human-delivered speech in domains such as news narration and film dubbing (Bonet, 2021). This growing indistinguishability has sparked existential anxiety among voice performers and students alike, casting doubt on the future viability of human artistic labor. For students pursuing voice acting in film and television, these technological shifts introduce a dual psychological burden: the fear of professional obsolescence and the intensifying demand to cultivate expressive authenticity in an increasingly digitized media environment. Traditional training methods prove insufficient amid this disruption, as the perceived “irreplaceability” of human expression must now be earned through emotionally resonant and creatively distinctive performance (Batchelor, 2019).

In this regard, voice performance—such as dubbing, vocal acting, and sound design—needs to be regarded as a fluid, time-based art form that functions both cognitively and affectively. The Vienna Integrated Model of Art Perception (VIMAP) provides a valuable framework for thinking about these dimensions in terms of its five categories of aesthetic outcomes: Facile, Novelty, Harmonious, Negative, and Transformative. Although initially designed for the perception of visual art, VIMAP’s structure can be applied to auditory experience as well, as both involve expectation, felt involvement, and reflective interpretation. Capitalizing on this cross-modal understanding, the current study combines VIMAP with an adapted Expressive Power Scale in a mixed-methods design to examine the interrelationship between AI-related anxiety and students’ expressive performance. The study is focused on answering three primary research questions: (1) How do voice arts students rate their own expressiveness in the presence of AI, and how do independent ratings differ? (2) What is the incidence of AI-induced anxiety among these students, and does it impact their expressive output? (3) To what extent is the gap between self and external judgments influenced by AI-related worries?

## 2 Literature review

### 2.1 Aesthetics and cognitive engagement in voice-based art

Voice-based art, as an interdisciplinary form, merges elements of music, visual arts, technology, and spatial design, creating a rich aesthetic experience rooted in perception, emotion, culture, and technological mediation (Lund and Anastasi, 1928; Pardo, 2017). Beyond singing and vocal performance, it includes diverse practices such as film dubbing, spoken-word poetry, audiobook narration, and sound design—each demanding distinct expressive skill (Carroll, 2012). As digital media technologies evolve, so too do the methods and sensibilities of voice art, prompting scholars to explore its cognitive and emotional dimensions from various disciplinary perspectives (Johnson, 2018; Cecchi, 2020). Aesthetically, voice art engages the interplay of sound, space, time, and the listener’s subjective perception, requiring artists to consider not only the acoustic and spatial characteristics of voice but also how these elements are interpreted across different listening contexts (Licht, 2021).

To analyze this complex aesthetic landscape, Chatterjee and Vartanian (2014) three-stage model—comprising perceptual analysis, emotional integration, and cognitive evaluation—offers a foundational approach to studying multimodal art. Building on this, Leder et al. (2004) Vienna Integrated Model of Art Perception (VIMAP) adds a nuanced five-stage process: Facile (surface perception), Novelty (interest and surprise), Harmonious (integration and coherence), Negative (dissonance), and Transformative (meaning reconstruction through reappraisal) (Pelowski et al., 2017). Although originally developed for visual arts, VIMAP’s structure aligns closely with the temporal and affective qualities of auditory and voice-based art. For instance, coherent narrative in vocal performance may reflect the Harmonious stage, while avant-garde sound art might catalyze Transformative experiences by challenging expectations (Ihde, 2007). In this study, VIMAP serves as a central theoretical framework for examining how students in voice performance programs navigate expressive development and aesthetic engagement within AI-augmented creative environments.

### 2.2 Performance competence in voice arts education

In the study of voice performance, evaluating expressive competence is vital for understanding and enhancing artistic communication. Whitehouse (2008) and Pardo (2017) emphasize that performance assessment should extend beyond technical precision to include emotional depth, a position further supported by Warhurst et al. (2017) and Latinus et al. (2011). Audience perception, meanwhile, plays a pivotal role in artistic success—particularly in voice-based professions where external evaluations significantly influence employability and reception (Shrivastav, 2006). Recent research highlights that expressive impact is shaped by an interplay of auditory cues, visual signals, speaker identity, perceptual modality, and cultural context (Campanella and Belin, 2007; Schiller et al., 2023; Miller et al., 2025), suggesting the need for a multisensory and culturally informed approach to performance evaluation.

To satisfy this requirement, researchers have created numerous tools to systematically evaluate expressive voice performance. Some of these are the Speech Quality Measurement framework, Multidimensional Scaling of Breathy Voice Quality (Shrivastav, 2006), and the Perceptual Evaluation of Voice Quality (PEVQ) by Kreiman et al. (1993), which, although originally designed for clinical purposes, provide useful information in artistic assessment. To these are added more comprehensive psychological measures such as the Berkeley Expressivity Questionnaire (BEQ) of which informed the construction of the Expressive Power Scale in Performing Arts—a multidimensional theory examining emotional transmission, technical control, and audience resonance. The scale has been used effectively in gauging expressive performance in theater, music, and dance (e.g., Shpyrka et al., 2021; Izountouemoi and Esteves, 2023). Through the combination of psychological concepts and performance analysis, the Expressive Power Scale provides an ecologically valid and all-encompassing assessment of artistic expressiveness—of particular importance in an age where human touch in expression is key to distinguishing creative output from that generated by artificial intelligence.

### 2.3 The challenge of AI-related anxiety in arts education

The accelerated development of artificial intelligence (AI) technologies has radically shaped numerous industries and brought about intricate challenges to arts education. Artificial Intelligence Anxiety (AIA) is one such challenge, a newly described psychological phenomenon that has increasingly taken center stage in creative learning contexts where artistic expression is intertwined with human–machine interaction (Clift et al., 2021; Christian et al., 2024; Wang et al., 2025). In those areas of expression like voice performance and audio arts, identity and individuality are very much of core importance. In such cases, AI-related fears are especially intense. Students generally worry that advanced AI technology like voice synthesis, robot dubbing, and generating sound effects may somehow erode their artistic truth and creative control (Eisenmann et al., 2025). At the same time, the rapid development of AI technology requires ongoing technical adjustments, which contribute to enhanced cognitive load, diminished motivation to learn, and loss of self-esteem (Xu et al., 2024; Bougdira and Al Murshidi, 2025).

Surprisingly, more recent research indicates that not all types of technological anxiety are negative; in an affirmative climate, moderate anxiety can even enhance learning and innovation. Research by Yetişensoy (2024), and Almogren (2025) indicates that such anxiety may serve as a driving force for motivating learners to develop competencies and find innovative solutions. Specifically, construction of the Artificial Intelligence Anxiety scale (AIA) brings both theoretical and empirical grounding to AIA in the context of learning environments. The AIAS unifies four dimensions—Learning Stress, Job Replacement Concern, Sociotechnical Blindness, and AI Configuration Anxiety—displaying that AIA is not a unitary fear but a rich psychological construct with challenges to cognition, identity, and technological fluency. Thus, resolving AI anxiety effectively becomes crucial not merely to protect the mental health of students but also to facilitate their expressive development and creative resilience under the canopy of intelligent, technology-rich learning.

### 2.4 Research gap

Existing studies have much improved our knowledge about aesthetic experience and artistic expressiveness by providing dimensional frameworks and assessment tools, while increased scholarly interest has been paid to the role artificial intelligence (AI) plays in human creativity and performance. Amidst these advancements, however, there has been an evident lacuna as to how AI-related aspects—especially AI-induced anxiety—are influencing students’ artistic expressiveness in learning environments. Particularly, little has been investigated on the connection between self-rated and externally rated expressive ability under psychological and technological stress of the AI age, and most research does not address how to assist voice performance students in building their expressive capacity in the process. To close this gap, the current research syntheses the Vienna Integrated Model of Art Perception (VIMAP), the Expressive Power Scale, and measures of the AI Anxiety field to analyze how students of voice performance perceive and express expressiveness. The research aims to generate new knowledge about how arts education can develop to address the cognitive, emotional, and career needs of students in ever more AI-integrated creative industries.

### 2.5 Research model

This study employs a multidimensional theoretical framework that integrates cognitive aesthetics, psychometric evaluation, and affective psychology to examine how artistic expressiveness in voice performance is shaped within AI-enhanced learning environments. At the cognitive level, the Vienna Integrated Model of Art Perception (VIMAP) serves as the foundation for understanding students’ aesthetic engagement, with its five outcome types—Facile, Novelty, Harmonious, Negative, and Transformative—adapted to reflect learners’ interpretative responses to voice performance tasks. To assess expressive development, a modified version of the Expressive Power Scale in Performing Arts is utilized, breaking down vocal performance into quantifiable dimensions and enabling structured evaluation of students’ artistic growth through project-based assignments. Complementing Self-Assessed Artistic Expressiveness (SAE) and Third-party-assessed Artistic Expressiveness (TAE), this tool offers a more objective view of expressive competence. The framework also incorporates theories of technological anxiety and media displacement to explore how perceived threats—such as AI replacement risk, heightened cognitive demands, and emotional discomfort—shape students’ engagement. By synthesizing these components, the framework provides a comprehensive lens for analyzing the influence of AI-related pressure on expressive outcomes and how AI anxiety mediates discrepancies between self-perception and audience appraisal.

**FIGURE 1:**
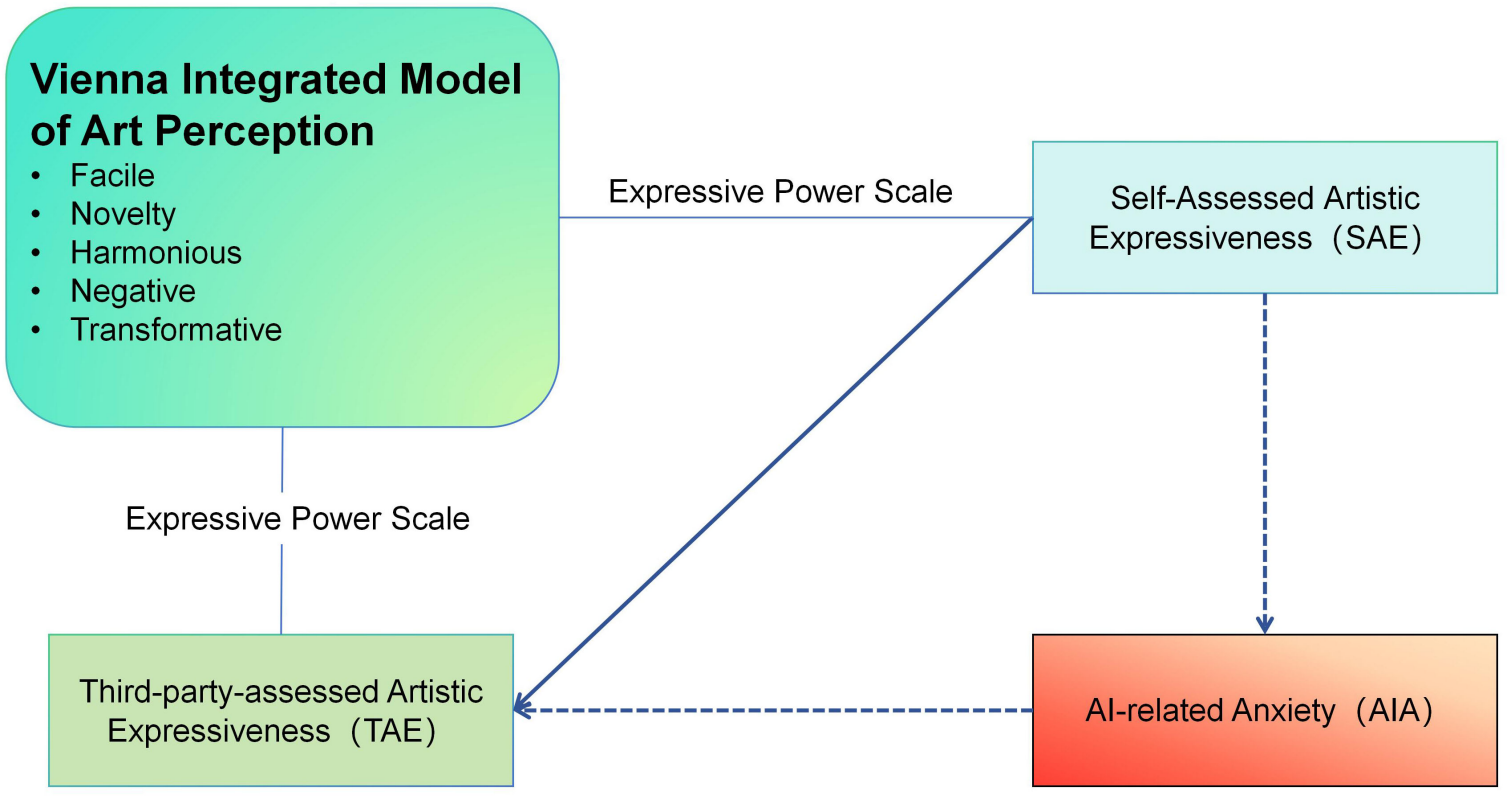
Integrated theoretical framework of Al anxiety and performance appraisal in voice arts education.

A complete visual representation of the research framework is provided in Figure 1.

## 3 Research methodology

### 3.1 Research design

This study adopts a mixed-methods research design, integrating quantitative surveys with qualitative interviews to provide a comprehensive analysis of how artificial intelligence (AI) impacts the development of students’ expressive abilities in voice performance. Quantitative data are collected through questionnaires administered to student participants and external evaluators. These instruments assess students’ perceived expressive ability, cognitive and emotional responses to AI, and performance quality ratings.

To contextualize and enrich the quantitative findings, semi-structured interviews are conducted with a subsample of students and instructors. These interviews explore participants’ learning experiences, perceived challenges, and reflections on the role of AI in the development of expressive skills in voice-based art forms.

### 3.2 Participants

This study comprised 828 valid participants from three Chinese universities (Institutions A, B, and C), including 414 undergraduate voice performance majors enrolled in the Dubbing and Artistic Performance course during the 2024-2025 academic year, and 414 external evaluators who independently assessed student-produced voice performances. Additionally, a subset of 10 participants (5 students and 5 instructors) was recruited for in-depth interviews to document their creative processes and affective experiences during AI-integrated project development.

### 3.3 Research instruments

This study employed three primary instruments—Questionnaire A, Questionnaire B, and a semi-structured interview protocol—to assess students’ expressive development and psychological responses to AI in the context of voice performance education.

Questionnaire A consists of three parts:

Part 1: Demographic Information

Participants provided information on gender, year of study, institution, artistic interest orientation, frequency of AI use, and whether AI tools were used in their submitted performance.

Part 2: Self-Assessed Artistic Expressiveness (SAE)

The SAE scale comprises five items that reflect students’ perceived artistic expressiveness. These items were adapted from the Vienna Integrated Model of Art Perception (VIMAP; Pelowski et al., 2017, 2020), which conceptualizes five outcomes of aesthetic experience—Facile, Novelty, Harmonious, Negative, and Transformative—and has been widely applied in empirical aesthetics research. To operationalize expressiveness in voice-based artistic performance, item development was further informed by the Self-Expression and Emotion Regulation in Art Therapy Scale (SERATS) and aligned with dimensions from the Expressive Power Scale (EES). The SAE scale in this study demonstrated good internal consistency (Cronbach’s α = 0.891).

Part 3: Artificial Intelligence Anxiety (AIA)

This section consists of four items measuring students’ cognitive and emotional responses to AI in artistic domains. The scale was adapted from the validated Artificial Intelligence Anxiety Scale (AIAS), which includes key domains such as learning burden, replacement anxiety, sociotechnical uncertainty, and affective discomfort. Items were modified to reflect the context of AI-mediated voice performance. The internal reliability of this scale in the current sample was high (Cronbach’s α = 0.865).

All questionnaire items were translated, back-translated, and pilot-tested with a representative student cohort to ensure clarity and cultural validity. A complete list of SAE and AIA items is presented in Table 1.

**TABLE 1:** Item list of self assessed expressiveness (SAE) and AI anxiety (AIA) questionnaire.

Construct	Item code	Item statement
Self-assessed expressiveness (SAE)	SAEQ1	I was able to complete the voice-over task naturally, with the vocal delivery generally matching the character.
	SAEQ2	When facing technical or expressive difficulties (e.g., timing, emotional delivery), I was able to adjust and complete the work effectively.
	SAEQ3	The creative process prompted me to reflect on and break through previous performance habits, leading to noticeable progress.
	SAEQ4	I experimented with new modes of expression or character design during this project, which made me feel excited and fulfilled.
	SAEQ5	I felt that my final work achieved strong coherence in emotion, character portrayal, and rhythm, resulting in an aesthetically harmonious experience.
AI anxiety (AIA)	AIA1	I am concerned that AI-generated voice technology may eventually replace human voice-over jobs in the future.
	AIA2	I worry that AI might be used to mimic or forge voice-over works using actors’ voices, including my own.
	AIA3	I feel compelled to invest considerable time learning AI tools just to stay professionally competitive.
	AIA4	I can’t fully explain it, but I find AI technology generally frightening.

Questionnaire B was designed for external evaluators (faculty and general listeners) to rate students’ performances on the same five VIMAP-derived dimensions as the SAE. The items in the TAE scale mirror those in the SAE, enabling direct comparison between self-assessed and externally assessed expressive outcomes. The TAE demonstrated strong internal consistency (Cronbach’s α = 0.904), supporting its construct validity as a third-party measure of expressive performance. Item wording was adjusted for observer perspective without altering underlying dimensions (see Table 2).

**TABLE 2:** Item list of the third-party artistics evaluation (TAE) questionnaire.

Construct	item code	Item statement
Third-party artistic evaluation (TAE)	TAEQ1	The voice-over matched the character design reasonably well and felt natural in presentation.
	TAEQ2	Although the performance appeared technically or expressively challenging, the final result demonstrated solid execution.
	TAEQ3	The work displayed a clear breakthrough in expressive technique or creative design compared to typical student projects.
	TAEQ4	The innovative aspects of the performance—such as voice timbre or dialogue design—caught my attention and interest.
	TAEQ5	The performance achieved strong alignment between voice, character, rhythm, and atmosphere, creating a sense of resonance and coherence.

The interview guide was constructed based on the VIMAP framework and key themes identified in technological anxiety literature (Almogren, 2025). It aimed to elicit qualitative data on learners’ perceptions of AI’s role in artistic expression, emotional responses to AI use, and strategies for managing performance-related challenges. The protocol included prompts covering themes such as novelty, creative autonomy, tool mastery, and emotional conflict see Table 3).

**TABLE 3:** Semi-structured interview protocol aligned with VIMAP aesthetic outcomes.

VIMAP dimension	Thematic focus	Interview questions
Facile	Initial technical and expressive matching	- Did you feel your voice matched the character naturally during the task? - Did AI tools help you improve clarity, tone, or alignment with the scene?
Novelty	Creativity and innovation	- Did you try any new expression techniques or character ideas? - Did AI tools help you discover novel ways to enhance your voice performance?
Harmonious	Emotional and structural coherence	- Did you feel your voice, rhythm, and emotional delivery worked together to form a coherent whole? - Did audience reactions reflect a sense of resonance?
Negative	Expressive or technical frustration; AI anxiety	- Were there moments when you felt stuck or uncomfortable during the project? - Did AI tools cause any confusion, frustration, or resistance during use?
Transformative	Reflective learning and artistic growth	- How has this project changed the way you think about voice performance? - Do you feel your expressive abilities have improved or evolved in any noticeable way?
AI attitudes	Perceived threat and identity concerns	- Are you concerned about AI replacing voice performers? - Do you see AI as a creative aid or a threat to your artistic identity?
Pedagogical insights	Learning experience and instructional support	- What part of the project or instruction helped you most? - What support would you like to receive in future AI-integrated voice courses?
Future orientation	Agency and identity in the AI era	- How do you imagine your role as a voice artist in an AI-integrated media environment? - What skills do you believe will remain uniquely human and irreplaceable?

### 3.4 Validity and reliability

The validity of the research instruments in terms of content is guaranteed through their firm theoretical basis and congruence with authoritative frameworks. Questionnaire A borrows from the Vienna Integrated Model of Art Perception (VIMAP) and the Expressive Power Scale to assess SAE, while its AI Anxiety (AIA) section is borrowed from proven scales on technological anxiety to guarantee appropriateness and construct clarity. Questionnaire B is a reflection of the SAE design to facilitate sound third-party assessment, with increased validity for comparative judgments. Pilot testing and expert review ensured item refinement by enhancing item clarity and thematic consistency and reinforcing content validity. For reliability purposes, both questionnaires use five-point Likert scales and randomized item order to enhance response consistency and minimize bias. Internal reliability was confirmed through Cronbach’s alpha pretesting, where all the subscales were well above the generally accepted value of 0.70. The combination of these measures confirms that the tools are not only valid in measuring the intended constructs but also reliable in providing consistent data among respondents.

### 3.5 Research procedure

The research procedure began with a preparation phase that involved developing and pilot-testing research instruments and integrating a Project-Based Learning (PBL) task into the voice performance curriculum. Students were organized into teams of 5–6 and guided to use AI tools to collaboratively complete creative dubbing projects for selected film or animation scenes, emphasizing reinterpretation over replication. Final outputs were uploaded to a university film culture festival webpage, where peers from other disciplines rated the performances. After project completion, Questionnaire A was administered to students to assess their self-perceived expressiveness and AI-related anxiety, while external viewers completed Questionnaire B to evaluate the artistic quality of each character’s voice performance, with each character rated only once per evaluator. Within a week, semi-structured interviews were conducted with selected students and instructors to capture in-depth qualitative perspectives on their experiences with AI and creative learning. Quantitative data were analyzed using SPSS to explore group differences, correlations, and predictive trends, while thematic analysis of interview transcripts through open coding identified key patterns. The results were synthesized along three focal dimensions: students’ cognitive and emotional responses to AI, discrepancies between self and third-party evaluations of expressiveness, and the impact of the PBL task on students’ voice-based expressive development.

## 4 Data analysis

### 4.1 Descriptive statistics

#### 4.1.1 Self-assessed artistic expressiveness

**FIGURE 2:**
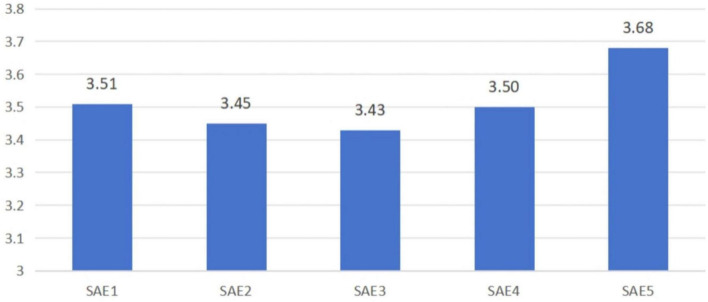
Mean scores for self-assessed artistic expressiveness (SAE).

Among the five items measuring SAE, SAE5 recorded the highest mean score (M = 3.676), while SAE3 had the lowest mean score (M = 3.430). Overall, these scores reflect a moderate level of self-perceived expressive ability among the student sample. Figure 2 displays the detailed results across the SAE dimensions.

#### 4.1.2 AI anxiety

**FIGURE 3:**
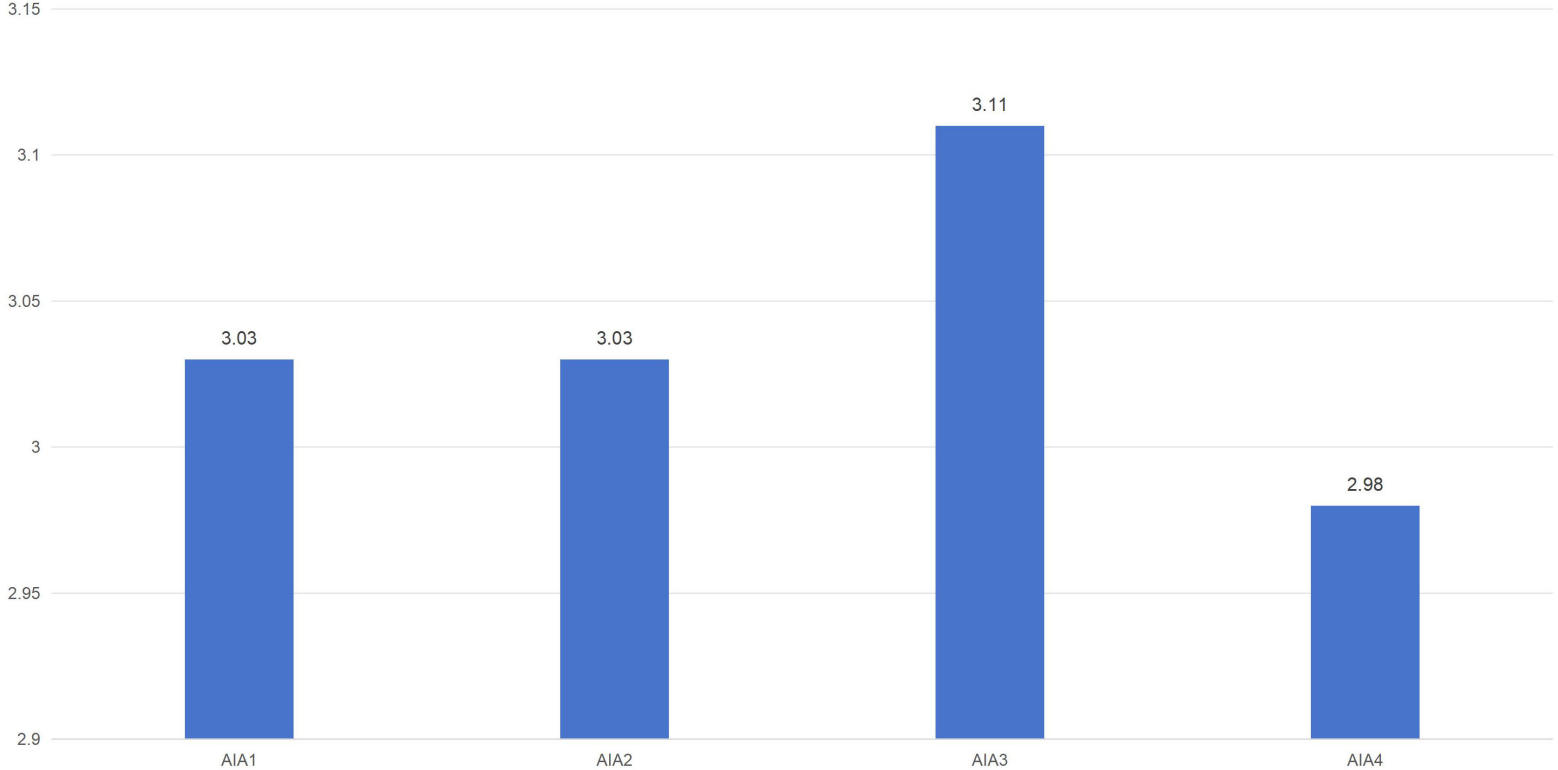
Mean scores for AI-related technological anxiety (AITA).

The AIA yielded a mean score of M = 3.11 with a standard deviation of SD = 0.902, indicating a moderate level of anxiety among students toward AI technologies in voice performance. Among the four items, AIA3 had the highest mean (M = 3.11), while AIA4 had the lowest (M = 2.98). Figure 3 illustrates the item-wise breakdown of AIA scores.

#### 4.1.3 Third-party-assessed artistic expressiveness

**FIGURE 4:**
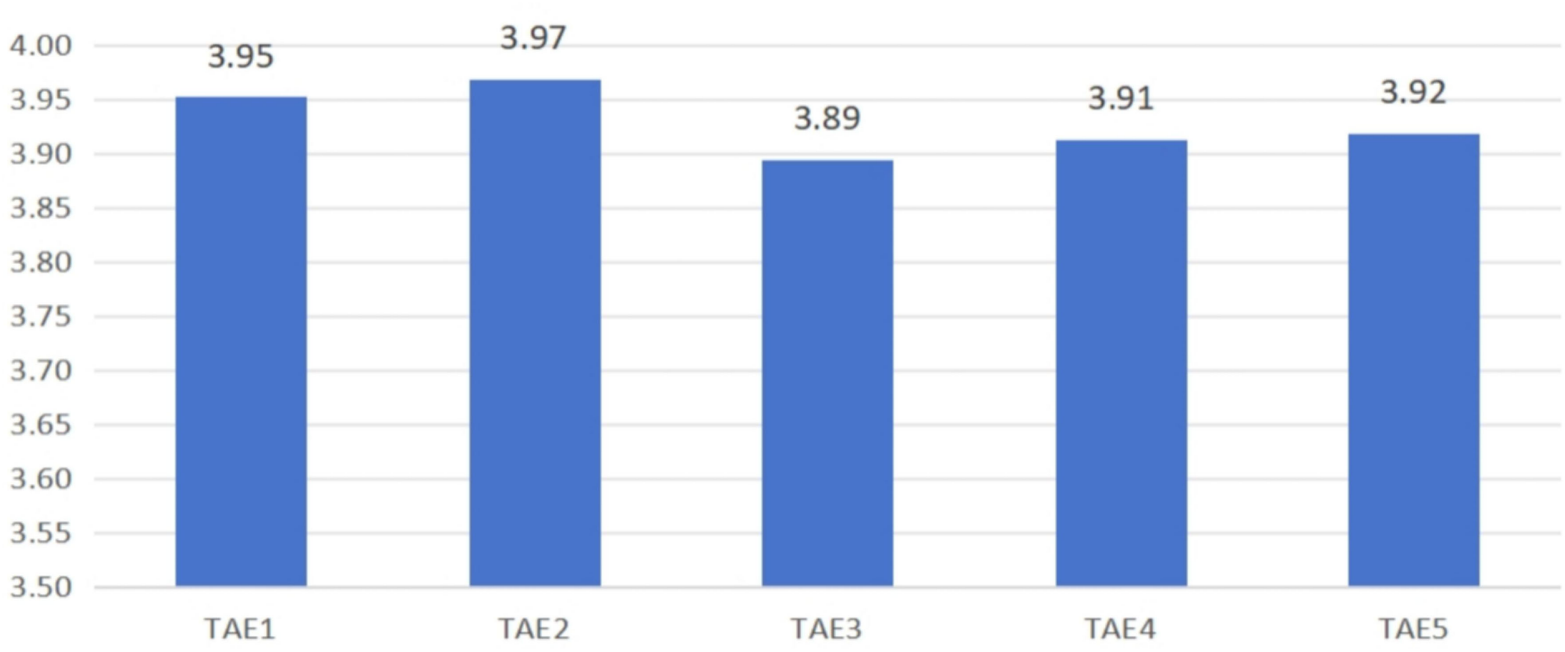
Mean scores for third-party-assessed artistic expressiveness (TAE).

The results from the external evaluator questionnaire (Questionnaire B) showed that the mean score for TAE was M = 3.930, with a standard deviation of SD = 0.701. These results suggest that external audiences generally rated the expressive quality of students’ dubbing performances more favorably than students rated themselves. See Figure 4 for the breakdown of TAE scores by dimension.

The Self–Other Rating Discrepancy (SOD)—defined as the difference between students’ self-assessments and third-party evaluations of expressive performance—had a mean value of -0.415 (SD = 0.748). This negative discrepancy indicates that students consistently rated their own expressive abilities lower than external evaluators did.

The largest discrepancy occurred in the Negative dimension (Challenge Coping), suggesting that students were particularly self-critical when evaluating their ability to handle performance difficulties. In contrast, the smallest discrepancies were found in the Harmonious and Novelty dimensions, indicating greater alignment between self- and other-ratings in areas related to aesthetic coherence and creative engagement.

**FIGURE 5:**
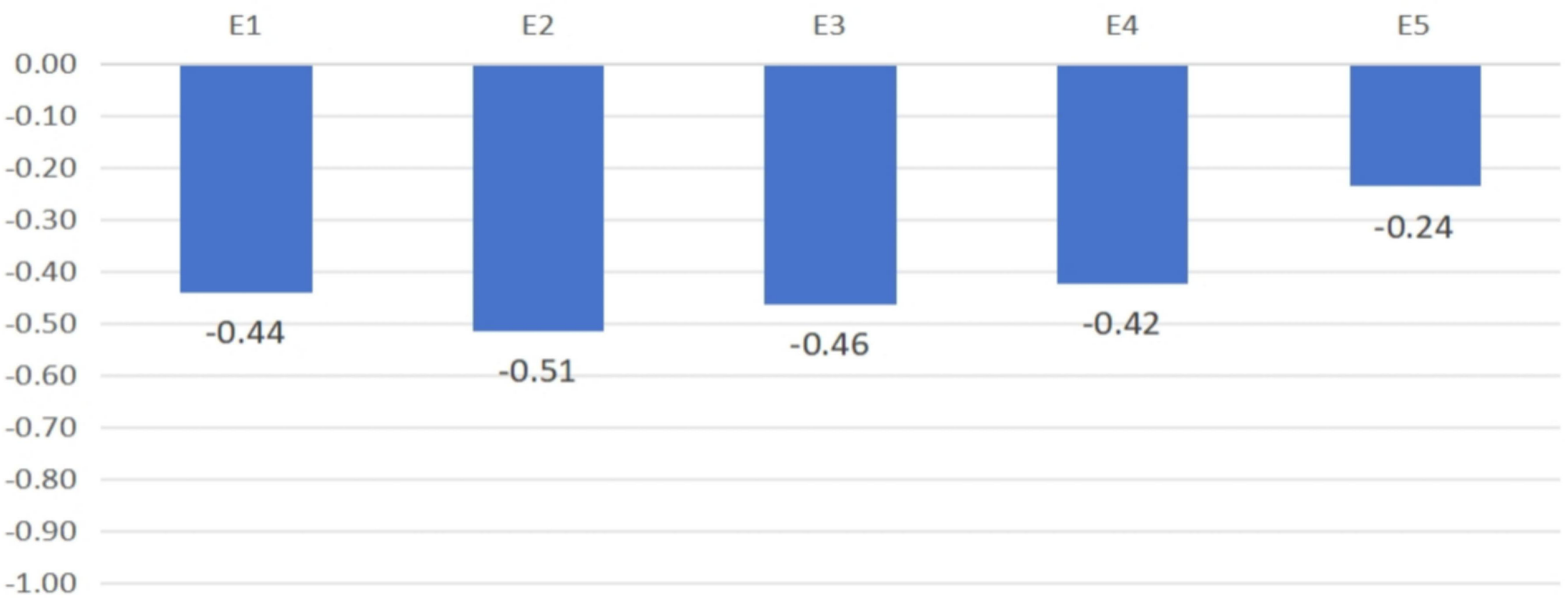
Self-other rating discrepancy (SOD) across VIMAP-based expressive outcome dimensions.

Figure 5 presents a visual comparison of the discrepancies across the five VIMAP-based outcome categories.

### 4.2. Correlational analysis

#### 4.2.1 Gender differences in expressiveness

An analysis of variance (ANOVA) revealed statistically significant differences in both SAE and TAE based on gender. Specifically, female students (coded as 1) reported and received higher mean scores on both SAE and TAE compared to male students (coded as 2) (see Table 4).

**TABLE 4:** Gender-based differences in SAE and TAE.

	Gender (mean ± SD)		*f*	*p*
	Female (*n* = 287)	Male (*n* = 127)		
D(5)	3.85 ± 0.69	4.11 ± 0.69	11.937	0.001**
A(5)	3.43 ± 0.90	3.71 ± 0.96	8.318	0.004**

**p < 0.01.

#### 4.2.2 AI usage frequency and expressive performance

Pearson correlation analysis demonstrated that frequency of AI usage was significantly correlated with all three core measures: AIA, SAE, and TAE. Higher AI usage was associated with lower anxiety and higher self- and other-assessed expressiveness (see Table 5).

**TABLE 5:** Correlations between AI usage frequency and key variables.

Variable	*r* (with AI usage frequency)
AIA	−0.259**
SAE	+0.501**
TAE	+0.640**

**p < 0.01.

#### 4.2.3 Item-level self–other rating discrepancy

An independent-samples *t*-test was conducted to examine the item-level differences between students’ self-ratings and third-party evaluations across the five SAE dimensions (see Table 6).

**TABLE 6:** Self–other rating discrepancy by dimension (SAE vs. TAE).

Item	Self (Mean ± SD)	Other (mean ± SD)	*t*	*p*
SAE1	3.51 ± 1.03	3.95 ± 0.80	−6.859	0.000**
SAE2	3.45 ± 1.13	3.97 ± 0.76	−7.691	0.000**
SAE3	3.43 ± 1.08	3.89 ± 0.84	−6.889	0.000**
SAE4	3.50 ± 1.05	3.93 ± 0.81	−6.477	0.000**
SAE5	3.68 ± 0.98	3.91 ± 0.82	−3.783	0.000**

**p < 0.01.

The greatest discrepancy was found in SAE2 (Transformative Experience) with *t* = −7.691, indicating substantial underestimation by students of their transformative potential.

The smallest discrepancy was in SAE5 (Harmonious Experience), with *t* = −3.783, suggesting relatively aligned perceptions between students and evaluators in this domain.

#### 4.2.4 Relationship between AI anxiety and SOD

Correlation analysis further revealed that students’ AIA was significantly associated with the degree of Self–Other Rating Discrepancy (SOD). All four AIA items showed moderate to strong negative correlations with SOD values, indicating that students with higher levels of anxiety tended to underrate their expressive abilities relative to external assessments (see Table 7).

**TABLE 7:** Correlation between AIA items and self–other rating discrepancy.

AIA item	*r* (SOD)	*p*
AIA1	−0.427*	0.000
AIA2	−0.366**	0.000
AIA3	−0.469**	0.000
AIA4	−0.376**	0.000

*p < 0.05, **p < 0.01.

#### 4.2.5 Non-significant factors

Other demographic variables—such as year of study, institution, and whether AI tools were used in the final project—did not show statistically significant correlations with SAE, TAE, or AIA scores.

### 4.3 Structural equation model data

Based on the proposed research framework, a structural equation model (SEM) was constructed to test the hypothesis that students’ AIA and SAE predict their TAE. The results confirmed this assumption.

The overall model demonstrated acceptable to good fit across multiple indices (see Table 8).

**TABLE 8:** Model fit indices for the structural equation model.

Fit index	Value	Threshold	Interpretation
χ^2^/df	3.325	<3–5	Acceptable
GFI	0.921	>0.90	Good
RMSEA	0.075	<0.08	Acceptable
RMR	0.030	<0.05	Excellent
CFI	0.968	>0.90	Excellent
NFI	0.955	>0.90	Excellent
TLI (NNFI)	0.960	>0.90	Excellent
AGFI	0.888	>0.90	Acceptable (near cutoff)
SRMR	0.033	<0.08	Excellent
RMSEA CI	0.065–0.085	–	Within acceptable range
AIC/BIC	60.811/185.613	–	Lower than null model

These results indicate that the model structure is statistically sound and provides a reliable basis for interpreting relationships among the latent variables. Key structural paths are summarized in Table 9.

**TABLE 9:** Standardized path estimates for the structural equation model.

Path	Standardized β	SE	*z*	*p*	Interpretation
AIA → TAE	−0.732	0.050	11.461	<0.001	Strong negative effect
SAE → TAE	+0.129	0.040	2.364	0.018	Weak positive effect

In addition, all items loading onto the latent constructs (AIA, SAE, TAE) showed strong standardized factor loadings, ranging from 0.801 to 0.941, confirming the internal consistency of the measurement model.

Students with higher levels of AIA tended to receive significantly lower ratings from external evaluators on their expressive performance (β = −0.732, *p* < 0.001). This suggests that anxiety toward AI may manifest behaviorally in the performance process, potentially diminishing expressive quality as perceived by others.

Students’ SAE was positively correlated with third-party evaluations (β = 0.129, *p* = 0.018), though the effect size was modest. This indicates some alignment between self-perception and external judgment, but also highlights the influence of emotional and psychological factors—such as anxiety—in mediating expressive outcomes.

### 4.4 Qualitative data

The qualitative data, derived from semi-structured interviews with students and instructors, were analyzed using thematic frequency analysis and coded according to the five outcome categories of the Vienna Integrated Model of Art Perception (VIMAP). Five key themes emerged, each corresponding to a distinct expressive outcome.

#### 4.4.1 Facile outcome: tone matching and surface-level execution

Participants frequently described their efforts to ensure that their voice aligned appropriately with the character’s visual and emotional profile. Many found AI tools helpful in refining technical aspects of their vocal performance. These responses reflect a reflecting surface-level technical precision, aligned with the positive correlations between frequent AI usage and both SAE (*r* = +0.501, *p* < 0.01) and TAE (*r* = +0.640, *p* < 0.01) as seen in Table 5, suggesting that repeated practice with AI tools enhanced basic expressive execution.

*“I found that AI helped me polish the audio and make my voice match the character more precisely.”*

*“In the PBL task, I realized that sound isn’t just sound. If I could show waveform effects visually, that would impress the audience—it’s really cool.”*

#### 4.4.2 Novelty outcome: excitement about new AI functions

Students expressed enthusiasm over discovering previously unknown AI features that enhanced creativity and expanded their expressive toolkit. These comments indicate novelty-driven learning, where AI tools sparked a sense of innovation and empowered artistic expression. This is clearly reflected in Table 7 which emphasized creative excitement with AI, resonated with relatively small self–other rating discrepancies (SOD) in the Novelty dimension (AIA = −0.42), indicating that both students and evaluators recognized the added value of innovative AI features.

*“This project encouraged us to actively explore AI. I had no idea it could add water sounds to classical poetry, hoofbeats to historical scenes, or even auto-subtitles for foreign characters. I was amazed!”*

*“One of the voice libraries I used let me transform my words into the speaking style of different people. That really helped me when writing new lines for creative characters.”*

#### 4.4.3 Harmonious outcome: aesthetic integration and expressive immersion

Students emphasized their desire to create emotionally resonant voice performances, often leveraging AI to heighten impact—but with varying results. This theme illustrates efforts to reach harmonious integration, where human authenticity and technological enhancement coalesce in aesthetic unity. It is evident in the findings in presented in Table 6, where it was found out that further support in the smallest SOD values (*t* = −3.783, *p* < 0.001), showing that aesthetic integration between human and AI input was perceived consistently by performers and external audiences alike.

*“Of course I want my voice to be emotionally moving! The AI sound effects were so explosive—but somehow the ratings didn’t reflect that, haha! Maybe I was too experimental?”*

*“I was a bit insecure about my use of AI, but our project scored well. Maybe it’s because my voice conveyed a kind of sincerity that AI still can’t replicate.”*

#### 4.4.4 Negative outcome: confusion, frustration, and identity anxiety

Several students conveyed frustration with AI’s limitations and a deeper concern about the future of human artistic identity in a machine-dominated environment. These responses reflect both technical frustration and existential artistic anxiety, characteristic of Negative outcomes. This is further supported by the findings in Table 6 as corresponded with the strongest self–other discrepancy in Challenge Coping (*t* = −7.691, *p* < 0.001), as well as with the SEM finding that AI anxiety exerted a strong negative effect on TAE (β = −0.732, *p* < 0.001).

*“Sometimes AI tools feel unusable—they don’t do what I want. But what really scares me is the thought of my voice being ‘stolen’ someday. If AI can already mimic celebrities, what happens when it can mimic me?”*

*“I used to know what audiences wanted. Now I’m not sure. Everyone’s using the same AI tools. Like, horror scenes always use the same ‘screeching door.’ That’s not creative. I tried to do something different—and it worked.”*

#### 4.4.5 Transformative outcome: integration, growth, and creative empowerment

Despite initial apprehensions, many students described a shift in mindset—from anxiety to strategic adoption—seeing AI not as a threat but as a professional tool for artistic growth. This illustrates the Transformative outcome, where learners integrate new tools into their identity as expressive artists. This was linked to significant underestimation of transformative potential by students (*t* = −7.691, *p* < 0.001), alongside qualitative evidence of mindset shifts from anxiety to empowerment as seen in Table 6.

*“AI is evolving so fast. It’s overwhelming. This project took all my spare time. But I can see it becoming part of my artistic toolbox.”*

*“I used to feel anxious—like I’d be out of a job after graduation. But now I feel more empowered. AI gives me more ways to create and more ways to bring my ideas to life. Overall, I feel optimistic.”*

## 5 Results

### 5.1 Students’ assess expressive ability and evaluated externally

Both qualitative and quantitative data reflect that students showed overall strong expressive ability in the voice acting project incorporating AI, according to self-assessments and external evaluations. See Figure 2 for the detailed results of the SAE. On the scale of SAE, the students expressed a high-to-moderate perception of expressiveness (M = 3.515, SD = 0.924), with the highest score for emotional resonance and congruence with character and setting (SAE5, M = 3.676) and the lowest for perceived improvement in dubbing skills (SAE3, M = 3.430). External assessors—professional teachers and non-professional audience members—also assessed students’ work as positive, with a general mean rating of M = 3.930 (SD = 0.701) on the TAE measure. The most highly rated statement recognized students’ capacity to overcome obstacles (TAE2, M = 3.97), with the lowest, although positive, concerning perceived technical advances (TAE3).

Statistical analysis also indicated that regular use of AI tools was strongly correlated with greater scores in both SAE and TAE measures, indicating that students who used AI tools more were in a position to attain better expressive performances. Interestingly, male students performed significantly better than female students for both self-assessment and independent evaluation, indicating possible gender-based differences in confidence, engagement, or familiarity with the tools. Corroborating these quantitative results, qualitative interviews also underscored the ways in which AI software facilitated students to hone their vocal tone, increase their sound libraries, try out varied audio effects, and gain more self-knowledge of their artistic potential—showing that AI was not just a technical tool but also a creative booster in the learning process.

### 5.2 Students experience AI-related anxiety, and it’s affect expressive performance

Data analysis revealed a moderate level of AI-related anxiety among students as presented in Figure 3. All four items on the AIA scored above the midpoint of 2.5. The most prominent concern was related to AIA4 (“I feel forced to spend significant time learning AI technologies in order to remain professionally competitive”).

Structural equation modeling (SEM) confirmed the strong negative impact of AIA on expressive performance as perceived by others. Specifically: Learners’ perceived AIA significantly and negatively predicted TAE, with a standardized path coefficient of β = −0.732 (*p* < 0.001). This finding suggests that students with higher levels of techno-anxiety tend to underperform in ways that are perceptible to external audiences, possibly due to reduced confidence, hesitation in vocal delivery, or emotional inhibition. Qualitative data provide further insights. Rather than worrying directly about AI replacing their jobs, many students expressed identity-based concerns, such as: Fear that AI could “steal” their voices or artistic uniqueness. Frustration that AI-generated dubbing is becoming homogeneous and predictable, thus undermining creativity. Concern about the escalating pressure to constantly acquire new technical skills just to keep pace with technological changes.

These findings suggest that AIA is not merely about job security but reflects deeper emotional and cognitive tensions around human agency, originality, and the evolving role of artists in AI-mediated environments.

### 5.3 AI anxiety influence the SOD

Analysis of the data showed that there was a moderate degree of AI-related apprehension among students, with all four items on the AIA exceeding the midpoint of 2.5, and that the most prevalent concern involved pressure to acquire AI technologies in order to stay professionally competitive as seen in Table 7. Structural equation modeling (SEM) proved that AI anxiety negatively impacted expressive performance rated by third-party assessors (β = −0.732, *p* < 0.001), suggesting that more anxious students were viewed as performing below average—most likely due to low confidence, vocal uncertainty, or emotional constraint. Qualitative data also deepened these impacts, with students’ fears less about losing their jobs and more about how they believed their artistic identity was being eroded. They were afraid that AI would “steal” their voice or render creative work into patterns of predictability. Many were also annoyed at having to constantly learn technical skills, which imposed mental weight and emotional pressure. Together, these results indicate that AI-related anxiety does not originate from merely extrinsic pressures but also from fundamental tensions surrounding human originality, creative authorship, and the evolving role of the artist in AI-encompassing spaces.

### 5.4 Pedagogical pathways emerge from students’ experiences

Semi-structured interviews were encoded according to the five outcome categories of Vienna Integrated Model of Art Perception (VIMAP) and assessed using thematic frequency analysis, yielding five of the themes that represent students’ experiences with AI-supported voice performance in a Project-Based Learning (PBL) context. The themes—skill building, creative autonomy, emotional engagement, technological issues, and future learning aspirations—shed light on changing learners’ perceptions. Instructionally, a number of important findings materialized: students who worked with AI tools indicated greater expressive self-awareness and confidence, although statistical measures failed to register a direct correlation between AI usage and performance ratings, further attesting to the pedagogical benefit of exposure and experimentation. But students also felt cognitive overload and stress when learning to apply unfamiliar tools, suggesting the necessity of guided support like vetted toolkits, technical instruction, and scaffolded activity. Moreover, several students expressed ethical concerns regarding AI in creative work, wondering if its potential exists to undermine human distinctiveness, standardize output, or steal individual artistic identity. These results imply the necessity of pedagogic approaches that not only encourage creative independence and technical proficiency, but also integrate critical literacy to enable learners to negotiate the sociotechnical aspects of AI in the arts—a point discussed later in the discussion.

## 6 Discussion

### 6.1 Understanding the role of AI through empirical aesthetic engagement

What our results show is that the employment of AI software greatly increases students’ felt expertise in expressive abilities in voice performance, frequently surprising students with how profound their own artistic development has been. This improvement can be meaningfully explained by using the Vienna Integrated Model of Art Perception (VIMAP) (Pelowski et al., 2017), providing a cognitive–affective approach to aesthetic growth. Project-Based Learning (PBL) activities—especially collaborative creation and iterative performance ones—seem to employ a series of VIMAP phases, including initial expectation mismatches, emotional disequilibrium, cognitive restructuring, and artistic insight. Students performing creative dubbing projects often described challenge-breakthrough cycles, consistent with the principle of metacognitive regulation in VIMAP.

Three interrelated mechanisms of expressive development were extracted from the data. Initially, Narrative Ownership was reinforced as students wrote and narrated their own new stories, increasing emotional investment while leaving the tech handling to AI software, freeing up creative bandwidth. Secondly, Exploratory Styling enabled students to play with different vocal tones and styles using AI integration, typically yielding more aesthetic balance and effect. Third, Reflective Breakthroughs occurred as a function of the PBL process where AI served as a stimulus for greater self-awareness and intentionality, allowing students to connect performance decision-making with expressive purposes. Collectively, these outcomes indicate that AI is not merely a technical booster but also a developmental accelerator, serving to enhance the expressive agency of students—a critical skill in the changing creative professions of the digital age.

### 6.2 Students’ cognitive and emotional responses to AI-induced anxiety

The study revealed that voice performance majors are moderately to highly anxious concerning the sudden upswing of AI-synthesized speech technology, fearing job replacement and the loss of creative control. Yet those who used AI tools more regularly documented lower anxiety, where the transition in perception was confirmed by both quantitative measures and qualitative observations. These students came to see AI as less of a threat and more of an accelerator of human expression, challenging them to redefine their roles as artists. Three main perceptual shifts were observed: from perceiving AI as a threat to cultivating interest in its possibilities; from helplessness to increasing creative independence through experiential engagement; and toward a future-directed artistic identity in which they perform not only as performers, but as designers and narrators of AI-enabled creative work. These results indicate that the incorporation of emotional resilience and AI fluency in arts education can enable learners to adopt new technologies as co-creatives, reframing their artistic identity in the digital era.

### 6.3 Perceived expressive features of student work: audience perspectives

Audience assessments indicated that voice performance students expressed significant expressive competence when aided by focused instruction and appropriate presentation media, with the Project-Based Learning (PBL) group consistently receiving superior ratings in emotional connection, story coherence, and creative staging. These findings indicate that organized, collaborative learning settings boost the expressive nature of student work since the PBL method allowed for strategic applications of AI tools for support tasks like voice modulation, script adjustment, and background editing, hence enabling students to have more time to concentrate on emotional delivery and audience influence. PBL group performances were often characterized as dynamic, assured, and varied, highlighting the merits of facilitated strategies in enabling students to balance human and technological resources. Additionally, consistent with previous research on AI performer bias, this study reaffirmed that though audiences might be fascinated by AI-powered enhancements, they tend to favor performances that express emotional sincerity, artistic purpose, and a human voice in particular.

### 6.4 SOD’s cognitive and affective mechanisms

A comparative study between third-party and student self-evaluations showed that AI-anxious students overestimated their expressive capacity consistently, even when their performances were well-received by instructors and mixed audiences. This psychological behavior indicates that apprehension about AI potentially replacing or reducing human vocal artistry may warp students’ self-perception and lead them to confuse technological ambiguity with personal insufficiency. The results show that techno-anxiety is detrimental to self-efficacy, exaggerating errors of creative ability doubt, especially among students with less exposure to AI tools. However, those who worked with AI in collaborative and supportive settings provided more realistic self-assessments and had lower levels of anxiety. This congruence between internal and external assessments most probably stems from both real enhancement of performance as well as lower internalized fear of technology substitution. These considerations highlight the need to integrate emotional resilience and digital literacy into expressive arts education, so that students have both technical proficiency and a secure artistic identity in the face of fast-changing technology.

### 6.5 Building effective pathways for cultivating expressive voice performance in the age of AI

In the era of artificial intelligence, developing expressive voice performance takes more than technical training—it demands psychological toughness, intellectual agility, and strategic flexibility. This research found two essential dimensions in realizing that goal. First, incorporating AI tools as creative collaborators rather than rivals greatly improved students’ expressive output. Most of the students indicated that AI-enabled dubbing enabled them to concentrate more on artistic delivery, while audiences enjoyed the novelty and flexibility that such incorporation represented. Nevertheless, even with the popularity of high-tech capabilities, scoring still preferred emotionally grounded human performance, highlighting the unique significance of affect in performance. Teachers are therefore urged to integrate AI tools into PBL-centered projects—not as time-savers, but as artistic elaborations—while keeping the emphasis on originality, emotional richness, and narrative coherence.

Second, the research established that anxiety related to AI had an unfavorable influence on students’ self-esteem and confidence, illustrating the significance of psychological preparedness in AI-based arts education. To remedy this, teachers must create a culture of open communication, critical inquiry, and scaffolding experimentation to make AI transparent and reimagine it as a tool for collaboration. Learners who had a clearer conception of AI as an aid formed more positive, exploratory orientations toward their art. Together, these results indicate an integrated model of AI-era arts education—one that integrates emotional acceptance, conceptual knowledge, and practical experience. Inserting these components into PBL structures can provide learners with the expressive skill and adaptive self-assurance required to excel as artists within a technologically enhanced creative environment.

Additionally, a key pain-point in the advancement of expressive voice work in the AI era is navigating the consequences of technology whilst maintaining ethical, legal, and artistic integrity. The rise in voice cloning technology raises questions of ownership, consent, and protecting the digital identity of the performer. State of the art voice modeling and data storage also raises legal and security concerns about privacy. In addition to structural issues, authenticity becomes an increasingly complicated question, as artificial modifications may cloud the distinct emotional and interpretative aspects of human artistry. The report findings confirm the necessity for the right conjunction of AI as a creative collaborator rather than an easy technological application of project-based learning (PBL). Although the voice dubbing influenced the student to focus on expressive vocally and broadened the expressive impulse, evaluators regularly ordered and praised those performances that were imbued with emotional truth proposing completeness, that authenticity and emotional truth matter when assessing artistic value. Therefore, effective pathways for voice pedagogy in the age of AI must both cover an institutional process for protecting voice and data identities, and cover pedagogical practices focused on original content and rich emotional interpretation, and psychological techniques to mitigate the risk of stress and anxiety when using AI tools that can distort reality. This fusion should ensure that learners develop both technical adaptability and expressive resilience pertinent to their artistic identity, as they navigate an increasingly digital performance environment.

## 7 Limitations and future directions

Although this investigation offers insightful contributions to the crossroads of artificial intelligence (AI), learning psychology, and aesthetics in voice performance pedagogy, a number of limitations should be noted. The participant group consisted of Chinese undergraduate students in elective voice performance courses, and because of this, the generalizability of the results to other educational or cultural environments may be limited. Relying on self-reported measures and single-timepoint assessments—albeit augmented with third-party ratings and interviews—embeds social desirability and unstable self-confidence biases. Moreover, external raters were not professionally trained, thus compromising objective aesthetic assessments. Unpredictability in the forms and intricacies of AI instruments utilized by pupils also makes data consistency more challenging due to differing interfaces and functionalities, which may generate different expressive results. Omission of a longitudinal aspect also restricts understanding of long-term impacts of AI incorporation on expressive skill acquisition and management of anxiety. Future studies must investigate cross-cultural contrasts, standardized AI usage, and randomized controlled trials to elucidate causal processes among AI usage, emotional reactivity, and artistic performance. Longitudinal studies monitoring expressive development through time, system-level comparisons between AI tools, and the incorporation of emotion-sensitive pedagogies such as reflective journaling or peer mentoring are also suggested to promote resilience and creativity in AI-augmented artistic learning.

## 8 Conclusion

This research investigated the effect of AI anxiety and use of AI tools on the expressive abilities of voice performance students in higher education using a mixed-methods design based on the Vienna Integrated Model of Art Perception (VIMAP), the Expressive Power framework, and technological anxiety theories. Self-assessment, external assessment, and interview findings showed that regular and intentional usage of AI tools—especially in project-based learning (PBL)—can have a beneficial impact on students’ expressive development. Students who used AI imaginatively scored higher in artistic self-knowledge and technical proficiency, and their performances tended to receive higher external ratings. In contrast, high levels of AI-related anxiety were also consistently linked to low self-confidence, greater self–other rating differences, and poorer expressive clarity, indicating that emotional reactions to technology may be a more significant hindrance to creative development than the technology itself.

Further, the research determined that although audiences valued digital augmentation and technical accuracy, they valued performances more that exhibited emotional sincerity and intentionality in storytelling—attributes based in human experience. These results highlight the importance of an integrated pedagogical framework that incorporates expressive skill development, AI literacy, and emotional strength. Incorporating these into coactive and reflective learning spaces can assist learners in coping with the double imperative of technological change and artistic integrity. Finally, as artificial intelligence continues to change the face of creative education, readying students for excellence in expression will involve not only training in performance skills but also resilience, critical thinking, and an entrenched sense of artistic identity.

## Data Availability

The datasets presented in this study can be found in online repositories. The names of the repository/repositories and accession number(s) can be found in the article/Supplementary material.
